# The Impact of “Vaping” Electronic Cigarettes on Spine Health

**DOI:** 10.7759/cureus.8907

**Published:** 2020-06-29

**Authors:** Brian Fiani, Christian Noblett, Jacob M Nanney, Neha Gautam, Elisabeth Pennington, Thao Doan, Daniel Nikolaidis

**Affiliations:** 1 Neurosurgery, Desert Regional Medical Center, Palm Springs, USA; 2 Osteopathic Medicine, University of New England, Biddeford, USA; 3 Medicine, University of Kentucky, Lexington, USA; 4 Neurology, Veterans Affairs Greater Los Angeles Healthcare System, Los Angeles, USA; 5 Pre-Medicine, Chapman University, Orange, USA; 6 Medicine, University of Texas Medical Branch, Galveston, USA; 7 Molecular and Integrative Physiology, University of Michigan, Ann Arbor, USA

**Keywords:** vaping, electronic cigarette, spine, smoking, electronic nicotine delivery systems, nicotine

## Abstract

“Vaping” or the use of electronic cigarettes (e-cigarettes) has greatly increased within the past decade, with growing popularity among adolescents. E-cigarettes have many harmful effects on multiple organ systems, but more research is needed to fully understand the extent of possible risks. Our narrative literature review aims to provide comprehensive insight into the impact of e-cigarette use on spinal health with a specific focus on intervertebral disc (IVD) health, bone health, and spinal fusion. There are many metallic compounds and chemical flavoring additives within e-cigarette liquids that are associated with human toxicity. These chemical toxins have been linked to increased oxidative stress leading to systemic inflammation. E-cigarette carcinogens have shown to have a toxic effect on osteoblast cells, and long-term use may decrease bone mineral density and increase the future risk for osteoporosis. Additionally, nicotine in e-liquids negatively impacts IVD health by creating hypoxic environments that degenerate the IVD vasculature and cellular matrix. While studies have demonstrated the inhibitory effects of nicotine use on spinal fusions in animal models, the impact of e-cigarette use on spinal fusion operations in human patients is currently lacking. Future research should focus on the influence of e-cigarette use on spinal health, particularly in adolescents with long-term follow-up, as childhood is a critical time for bone growth and development. Additionally, studies exploring the effects of e-cigarettes on spinal surgery outcomes, such as spinal fusions, are sparse in the literature. Further prospective research studies with a focus on the variety of e-cigarette chemical toxins and flavoring agents is needed to assess the impact on spinal health.

## Introduction and background

Electronic cigarettes (e-cigarettes) were first designed by a Chinese pharmacist in 2003 [1]. In 2006, e-cigarettes were introduced to the United States market as a smoking cessation tool marketed as a safer alternative to traditional cigarettes [2]. According to a recent study of U.S. adults, 95% of those interviewed believed e-cigarettes to be “cleaner and healthier” than conventional products, suggesting the validity of introductory claims were widely accepted by the general public [3]. The result was exponential growth in popularity among traditional combustible cigarette smokers, who represent 64.7% of e-cigarette users [4]. In a survey of approximately 15,000 U.S. adults between 2010 and 2013, e-cigarette use increased from 1.8% to 13%, approximately a seven-fold increase [4]. Electronic nicotine delivery systems (ENDS) evolved from traditional disposable e-cigarettes, which are similar to conventional cigarettes in flavor and appearance, to large-size tank devices, and finally “pod-mod” devices such as JUUL® [2]. The JUUL® was introduced in 2015, and immediately gained popularity amongst the youth due to its sleek design, desirable flavors, and the ability to smoke discreetly in prohibited locations [2]. The usage of ENDS has increased in young adults from 1.5% in 2011 to 20.8% in 2018 [2].

Initially, due to a lack of knowledge on the long-term toxicological effects of usage, opposition against e-cigarette health claims remained scant. In August 2019, hospital admissions for patients with lung injuries associated with e-cigarettes spiked, reaching a peak of 237 patient admissions on September 15, 2019 [5]. This highlighted the need for further research to broaden the understanding of risks associated with e-cigarettes. According to the Centers for Disease Control and Prevention (CDC), vitamin E acetate, a common additive in e-cigarette devices, represents the strongest link to e-cigarette, or vaping, product use-associated lung injury (EVALI) outbreak [5]. Evidence is not yet sufficient to rule out the harmful effects of other additives. Additional research is necessary to find correlations between specific generations of e-cigarette devices and the risks associated with each. These risks could include, and would not be limited to, cardiovascular, pulmonary, and immune systems, often presenting as inflammatory responses [1]. Potential harmful effects to other organ systems cannot be ruled out, as these systems need to be further analyzed. This review is intended to broaden the understanding of risks associated with e-cigarette use by analyzing the impacts on spinal health. The extent of the potential risk will be assessed by identifying harmful factors related to e-cigarette use that may impact intervertebral disc (IVD) health, bone health, and spinal fusion.

## Review

The molecular toxicology of vaping

The general consensus is that e-cigarette vapor has substantially lower levels of carcinogens and toxins found in traditional cigarette smoke. For example, Tayyarah et al. report that cigarette smoke contains between 3069 and 3350 μg/puff of the 55 harmful and potentially harmful constituents (HPHC) measured in the study [6]. Conversely, e-cigarette vapor contained <2 μg/puff of HPHCs, or ~99% less analytes than cigarettes [6]. However, there are novel toxicology risks associated with e-cigarettes that must be further evaluated. E-cigarettes have heating coils that are made of various metallic compounds and can be used with thousands of different e-liquids with various chemical flavoring additives [7]. Williams et al. quantified the abundance of metallic elements in various e-liquids using induced coupled plasma-optical emissions spectroscopy (ICP-OES) [8]. The authors reported that e-cigarette vapor contained aerosol particles >1µm of tin, silver, iron, nickel, aluminum, silicate [8]. Strikingly, nine of the 11 metals detected in e-cigarette vapor were equal to or greater than the concentration detected in cigarette smoke [8]. All of these metals are associated with human toxicity at high doses, particularly in the nervous, renal, and respiratory systems [8].

E-cigarette toxicity is largely affected by the flavoring of the e-liquid. There are over 7,700 commercially available e-liquids, most of which have not been tested [9]. Sassano et al. developed a high-throughput screening assay to study the chemical constituents of thousands of e-liquids using gas chromatography-mass spectrometry (GC-MS) techniques [9]. The authors found that e-liquids have large variations in toxicity depending primarily on flavoring additives [9]. In particular, the addition of vanillin (vanilla flavor) or cinnamaldehyde (cinnamon flavor) resulted in the highest levels of in vitro cellular toxicity [9].

Recent data by Crotty Alexander et al. suggests that e-cigarette exposure triggers an inflammatory response that results in cellular damage to the airways, specifically by disrupting pulmonary epithelial barrier function [10]. In a feed-forward manner, disruption of barrier function and continued exposure to chemical toxins found in e-cigarette vapor contributes to systemic inflammation that can result in downstream organ pathologies such as renal fibrosis [10]. An essential component of inflammation is oxidative stress. Oxidative stress, which is generated by increased levels of reactive oxygen species (ROS), can activate the transcription of inflammatory genes such as IL-8, a potent chemokine that recruits leukocytes and is involved in chronic inflammation [11]. Several groups have clearly demonstrated the link between vaping and oxidative stress using both in vivo and in vitro laboratory models. Lerner et al. measured intracellular glutathione (GSH) levels in mice exposed to e-cigarette vapor for three days (five hours total exposure time) [11]. GSH is an essential endogenous antioxidant that maintains cellular redox balance by responding to ROS stress [11]. The authors found that there was a significant decrease in GSH levels in mice exposed to e-cigarette vapor compared with air-exposed controls (P < 0.05) [11]. Additionally, there were modulations in the balance between the reduced and oxidized forms of GSH in mice exposed to e-cigarette vapor [11]. Ganapathy et al. found that human epithelial bronchial cells exposed to e-cigarette vapor for two weeks in vitro had significantly increased levels of oxidative damage, indicated by 8-oxo-dG DNA lesions, compared to the control group of air-only (P < 0.05) [12]. Cells exposed to e-cigarette vapor showed significantly increased levels of oxidative damage compared to cells exposed only to cigarette smoke (P < 0.05) [12]. Moreover, the authors reported a significant decrease in total antioxidant capacity in cells exposed to either e-cig vapor or cigarette smoke (P < 0.05), with no significant difference between the two experimental groups [12].

Primary research suggests that the principle mechanism behind physiological consequences related to e-cigarette exposure is oxidative stress and inflammation. Oxidative stress and inflammation are intricately linked bidirectional processes that together lead to cellular and tissue damage [13]. E-cigarette vapor itself, like cigarette smoke, is a potent source of ROS [14]. When using an e-cigarette, ROS are directly inhaled into the lungs and play an integral role in stimulating inflammatory signaling cascades [14,15]. Moreover, inflammation stemming from exposure to toxic chemicals in e-cigarette vapor results in the recruitment of immune cells like macrophages and the release of pro-inflammatory cytokines. Subsequently, inflammatory proteins and immune cells generate ROS that work in a feed-forward manner to increase inflammation and cellular damage, which can have widespread effects on various organ systems.

Impact on IVD health

An e-cigarette solution containing nicotine can impact IVD health through the degeneration of both vasculature and nucleus pulposus and annulus fibrosus cells. Iwahashi et al. supplemented a model organism with nicotine levels equivalent to that of a heavy smoker and demonstrated degenerative vascular changes around IVDs [16]. They showed both a decrease in density of vascular buds at the vertebral endplate, as well as, a reduction in the diameter of vascular lumen encircling the disc. They conclude the hypoxic environment facilitates the reduction of metabolism potential and ultimately leads to disc degeneration. Elmasry et al. similarly show that nicotine causes vasoconstriction leading to decreased glycosaminoglycans (GAG) in the IVD interior [17]. They also describe the down-regulatory effects of nicotine on GAGs in the cartilaginous endplate. The combined effects of both lead to IVD degeneration.

In other studies focusing on the cell architecture of the IVD, Kim et al. show that nicotine inhibits the production of GAGs and type II collagen in nucleus pulposus cells through the antagonistic effects on bone morphogenic protein 2 (BMP-2) [18]. Akmal et al. discovered a significant reduction in the ability of nucleus pulposus cells to proliferate and synthesize the extracellular matrix. They displayed that nicotine causes a dose-dependent degeneration of the IVD cellular matrix [19]. More recently, Nakahashi et al. support Kim et al. by exhibiting nicotine levels similar to that of smokers induce chondrocyte apoptosis within the vertebral endplate [20]. This was followed by distortions of the nucleus pulposus architecture due to decreases in type II collagen and proteoglycans. In all, a research review of nicotine, a substance present in e-cigarette solutions, shows the deleterious effect it has on the IVD health through morphogenic changes in vasculature and nucleus pulposus and annulus fibrosus composition.

Impact on bone health

Due to the growing worldwide popularity of “vape” (electronic cigarette) products advertised as a healthier alternative to tobacco, there is an increasing concern over health risks that are linked to vape use and how it affects bone health. Proper bone development is crucial to paving the way for the future directionality as to how the human body will grow. Unfortunately, when bone health is affected, the outcomes are adverse and can become chronic.

There are many triggers that affect the future potential of having healthy bones: from something as minute as a gene replication within the bone-forming osteoblast cells, to frequent exposure to polluted environments due to vapes. Vapes contain an electronic liquid (e-liquid) chamber that withholds an assortment of toxic chemicals such as cadmium, nicotine, and many other carcinogenic chemicals that invade the human body with oxidative stress. A study done in Sweden looked at cadmium to observe how detrimental this chemical can be on the longevity of the lifespan of a cell [21]. This study showed results that suggested negative effects with just low-level cadmium exposure to bones [21]. Along with cadmium, nicotine and other e-liquid flavoring agents could negatively alter osteoblast proliferation, differentiation or matrix deposition. In a recent study, researchers were able to demonstrate that smoking conventional tobacco leads to reduced bone mineral density and increased risk for osteoporotic fractures [22].

Many experts are especially concerned about minors using vape because the flavoring agents make them attractive to adolescents. Therefore, to understand the risk factors that are related to vaping is germane. Childhood is a critical time for optimal bone growth and development because approximately 90% of bone mass is accrued by early adulthood at about 18 years of age [23]. Therefore, Otero and her team took a deeper look into the exposure of e-liquids along with vape flavors and the osteotoxicity rankings of each individual flavor [24]. In this study, Otero mainly focused the attention towards human MG-63 and Saos-2 osteoblast cells that her team incubated for 48 hours with 0.004%-4.0% of commercially available e-liquids with and without nicotine [24]. Otero’s study had many key assessments as to how vaping can cause changes in cell viability and osteoblast markers [24]. From Otero’s research, they were able to ascertain the drastic effects on Saos-2 cells from the chemicals found in vapes even more so than with the MG-63 cells [24]. Another important finding they were able to conclude was that several vape containing e-liquids were highly cytotoxic at just 2% volume or higher [24].

Looking into the grouping of e-liquids further illustrated that the most highly potent e-liquid to influence bone growing osteoblastic cells was cinnamon e-liquid and the least cytotoxic were the flavorless vapes [24]. Another study took a step deeper into the cinnamon flavor vape and aerosol products to see how it affects MG-63 cells and found this osteoblast cell is sensitive to both e-liquids and aerosol condensates concluding that cytotoxicity of cinnamon-flavored e-liquids might be associated with oxidative stress [24,25]. This in vitro study was able to provide insight into how destructive cinnamon e-liquid is to a cell’s longevity by reducing cell viability and increasing ROS [24,25]. Moreover, they noticed a trend that cytotoxicity occurred independently of nicotine. Thus, many research experts are suggesting that flavored vape do have cytotoxic effects and these effects occur independently of the presence of nicotine, signifying that flavoring agents alone will induce cellular damage, on top of the well-known risks of nicotine consumption [26].

Now that we are certain that the ingredient of e-liquids in vapes is highly carcinogenic to humans, what does it mean to have insufficient bone health growth and what are the long-term effects from this? One common disorder is osteoporosis. Decreased bone mineral density increases the risk for development of osteoporosis [27]. Recent studies showing in vitro and in vivo data suggesting that the ingredients used in vape e-liquid are adverse to the body producing healthy bone structures that will provide support over the longevity of one's lifespan. Apart from osteoporosis, epidemiologists have reported that with the contact of vape chemicals, such as toxic metals, may promote the development of other musculoskeletal diseases such as rheumatoid arthritis (RA), and osteoarthritis (OA), among others [28]. In their study, epidemiologists found that the accumulation of cadmium in the tissues and blood of smokers has been related to the development of some musculoskeletal diseases [28]. It is clear that the invasive process of how vapes operate in the human body through the specific cell to cell interaction as well as how it can affect one’s bone health overall and lead to chronic medical diagnoses are factors that heavily support a trend for a compounding effect from vape e-liquid on bone health. Nevertheless, further research is necessary to look at the different parameters of how vape products are affecting bone health and better understand the life span of the osteoblastic type cells MG-63 and Saos-2 when one vapes or even through the contaminants found in air through exposure to second-hand vape chemicals.

Impact on spinal fusion

It is well documented that smoking cigarettes increases the risk of pseudoarthrosis in patients undergoing operative spinal fusions. A review by Berman et al., in 2017, established that smoking cigarettes increases the rate of perioperative complications associated with spinal fusion surgeries from both biochemical and clinical perspectives [29]. E-cigarettes have been promoted as a “healthier” alternative to smoking cigarettes in recent years. Although the negative effects of cigarettes on spinal fusions are well known, the effects of e-cigarettes on spinal fusions are not well documented.

The outcome and results of spinal fusion operations in patients who admit to e-cigarettes use are poorly described in the literature. A 2019 review by Amaro et al. provided a review of available knowledge on the effects of vaping on orthopedic surgeries that are pertinent to spinal fusions [30]. While the effects of e-cigarettes use on spinal fusion is not well documented, experiments of posterolateral spinal fusions in the rabbit model demonstrate that nicotine alone can affect surgical outcomes [30]. A 1995 study by Silcox et al. demonstrated systemic nicotine significantly increased pseudoarthrosis rate to 100% compared to 44% in control animals in a rabbit model of posterolateral spine fusion [31]. A 2000 study by Theiss et al. revealed nicotine alone inhibits the expression of multiple cytokines with a variety of functions during spine fusion [32].

While these studies point to the negative impacts of nicotine on spinal fusion, more recent studies provide evidence that low doses of nicotine may improve surgical outcomes. A 2015 study by Daffner et al. indicated the effects of nicotine on the rate of posterolateral spinal fusion may be dose-dependent in a rabbit model [33]. This study found that low doses of nicotine exposure may enhance spinal fusion, while high doses appear to be inhibitory [33]. While the effects of nicotine on spinal fusion outcomes have been studied in animal models, e-cigarette vapors contain an assortment of other chemicals that may impact surgical outcomes. Literature pertaining directly to the surgical outcomes in patients admitting to e-cigarette use is sparse. Further studies investigating exposure to the variety of chemicals in e-cigarette vapors and its impact on the surgical outcomes are necessary to definitively determine the direct impact of e-cigarettes on operative spinal fusion outcomes and results.

## Conclusions

E-cigarette use has greatly increased in the United States as an alternative to traditional cigarettes within the past years. Since 2015, changes in design and the addition of flavors resulted in increased popularity among adolescents especially. While many studies report the risk of harmful compounds in cigarettes, there are thousands of commercial e-liquids available and most are untested for toxicity. Our narrative literature review shows there are many potentially harmful effects of e-cigarette compounds and additives, but there is an additional need for prospective randomized controlled trials with long-term follow-up to definitively determine the impact on spinal health in regard to bone health, IVD health, and spinal fusion. Research shows exposure to some e-cigarette carcinogens have been associated with increased oxidative stress and inflammation. This oxidative stress has a negative impact on bone health by modifying osteoblast proliferation and differentiation. Nicotine in e-cigarette liquids has also been shown to degenerate IVDs by changing cell composition and vasculature. While studies have shown a relationship between traditional cigarette use and spinal fusion outcomes, research investigating the effect of e-cigarette use on spinal fusions is deficient in the literature. Further research is needed to evaluate the novel toxicology risks associated with the various compounds and flavoring additives used in e-cigarettes and their impact on spinal health.
